# Using an extended treatment regimen of lanadelumab in the prophylaxis of hereditary angioedema: a single-centre experience

**DOI:** 10.1016/j.waojou.2022.100664

**Published:** 2022-07-12

**Authors:** Mohamed Abuzakouk, Omar Ghorab, Hamad Al-Hameli, Fulvio Salvo, Deepa Grandon, Marcus Maurer

**Affiliations:** a Allergy and Immunology Department, Cleveland Clinic Abu Dhabi, Abu Dhabi, United Arab Emirates; b School of Medicine, Royal College of Surgeons in Ireland, Bahrain, Bahrain; c Fraunhofer Institute for Translational Medicine and Pharmacology ITMP, Allergology and Immunology, Berlin, Germany; d Institute of Allergology, Charité – Universitätsmedizin Berlin, Corporate Member of Freie Universität Berlin and Humboldt-Universität zu Berlin, Berlin, Germany

**Keywords:** Lanadelumab, Hereditary angioedema, Prophylaxis, Real-life

## Abstract

**Aim:**

To explore and compare the efficacy of standard (300 mg every 2 weeks) and extended (300 mg every 4 weeks) dosing regimens of lanadelumab for long-term prophylaxis of hereditary angioedema (HAE).

**Methods:**

We conducted a retrospective chart review of all patients with HAE on lanadelumab, which identified a total of 9 patients: 5 females and 4 males. The median age of patients was 31 years (IQR 20.7). The mean number of attacks per month before starting lanadelumab was 5.9 (SD 6.3). Patients were started on 300 mg of lanadelumab subcutaneously, every 2 weeks (standard group, n = 5) or every 4 weeks (extended group, n = 4).

**Results:**

We observed a statistically significant improvement in the number of angioedema attacks per month in all 9 patients (*p* = 0.007). Five out of 9 patients (56%) achieved complete remission from attacks after starting lanadelumab. The effect of lanadelumab on number of angioedema attacks was significant in both groups; extended group (*p* = 0.03) and standard group (*p* = 0.01).

**Conclusion:**

Lanadelumab is a safe and effective agent for long-term prophylaxis of HAE. An extended dosing regimen was equally effective as prophylaxis compared to a standard regimen. Further studies are needed to compare the 2 regimens in a larger patient group.

## Introduction

Hereditary angioedema (HAE) with C1 inhibitor (C1-INH) deficiency is a rare autosomal dominant disease characterized by painful, recurring attacks of swelling involving subcutaneous and/or submucosal tissue throughout the body.^1^ This is caused by mutations in the *SERPING1* gene, which codes for the serine protease inhibitor C1-INH, a key regulator of plasma kallikrein in the kallikrein-kinin cascade. HAE-driving *SERPING1* mutations can either lead to reduced levels of C1-INH (Type I), or dysfunctional activity (Type II). As a result, there is overproduction of kallikrein, subsequently leading to an overproduction of bradykinin, which causes the painful attacks of swelling that characterize the disease.^1^ The disease burden on HAE patients is heavy. In addition to the impairment in quality of life during the attacks of swelling, patients are also affected between attacks, due to the unpredictable nature of the disease, thus affecting daily activities at school, work, and home. Moreover, the disease bears a risk for severe and fatal outcomes, due to the potential for upper airway involvement.

The treatment of HAE with C1-INH deficiency involves on-demand treatment and prophylactic.^2^^,^^3^ Currently approved and recommended first-line prophylactic medications include C1-INH (intravenous or subcutaneous) and the plasma kallikrein inhibitors lanadelumab and berotralstat.^4^ Attenuated androgens (eg, danazol), introduced as HAE treatment in the 1960s, are now second line prophylactic agents as they cause unwanted side effects in most patients and therefore require close monitoring.^5^ Anti-fibrinolytics (eg, tranexamic acid) are no longer recommended for long-term prophylaxis.^4^ They can be used where first- and second-line agents are unavailable or contraindicated. While androgens and tranexamic acid are still commonly used in some countries, they are less safe and effective for preventing HAE attacks, compared with modern treatments.^6^

Recently, lanadelumab, a kallikrein inhibitor monoclonal antibody, was approved for the long-term prophylaxis of both type I and type II HAE with C1-INH deficiency in patients 12 years and older.^7^ Real-life reports have shown that it is a safe and effective prophylactic agent, however treatment cost is high.^1^^,^^8^^,^^9^ Using an extended dosing regimen (every 4 weeks) may prove helpful in difficult-to-treat patients who cannot afford the standard regimen (every 2 weeks). We report a single-center experience to compare the effectiveness and safety profile of an extended dosing regimen, compared to a standard regimen, of lanadelumab in HAE.

## Methods

Our patient group consisted of a total of 9 patients: 5 females and 4 males (Table 1). The median age of patients was 31 years (IQR 20.7). All patients had a confirmed diagnosis of HAE based on extensive laboratory investigations and a positive family history prior to starting treatment. Seven patients were diagnosed with type II disease; whereas 2 patients were diagnosed with type I disease. All patients were on long-term prophylactic therapy prior to treatment with lanadelumab. Five patients were on tranexamic acid, 3 were on danazol, and 1 patient was receiving regular intravenous plasma-derived C1-INH concentrate (Table 1). All patients had access to plasma-derived C1-INH concentrate for acute attacks. The mean number of attacks per month before starting lanadelumab was 5.9 (SD 6.3). Attacks were abdominal and cutaneous in all patients. Only 1 patient had a history of laryngeal attacks. All patients discontinued their previous prophylactic agent when lanadelumab was initiated. All patients received 300 mg of lanadelumab subcutaneously. Dosing frequency was dictated by disease severity, which was measured using the number of angioedema attacks per month.^10^ A cutoff baseline value of 4 attacks per month was used to allocate patients to 2 treatment groups: standard treatment group (lanadelumab every 2 weeks, n = 5), and extended treatment group (lanadelumab every 4 weeks, n = 4).

**Table 1:** Baseline characteristics of patients on lanadelumab

Patient	Gender	Age	Weight (kg)	HAE type	Type of prophylaxis
1	M	31	101	II	Danazol, 200 mg bd
2	F	42	114	II	C1-INH, 1000 units every 4 days
3	F	24	44	I	Tranexamic acid, 1 g tds
4	F	45	57	II	Tranexamic acid, 1 g tds
5	M	21	58	II	Danazol, 200 mg bd
6	F	37	65	I	Danazol, 200 mg bd
7	F	55	101	II	Tranexamic acid, 1 g tds
8	M	34	64	II	Tranexamic acid, 500 mg tds
9	M	41	117	II	Tranexamic acid, 500 mg tds

## Results

Patients were treated for a median number of 36 weeks (IQR 24). We observed a statistically significant improvement in the number of angioedema attacks per month in all 9 patients after 1 month of treatment (*p* = 0.01), 2 months (*p* = 0.01), 3 months (*p* = 0.009), and overall at the end of the study period (*p* = 0.007). Five out of 9 patients (56%) achieved complete remission from attacks after starting lanadelumab (Table 2). When taking the treatment groups individually, the effect of lanadelumab on number of angioedema attacks was significant in both groups; four-week group (*p* = 0.03) and two-week group (*p* = 0.01). Additionally, there was a statistically significant improvement in the number of attacks requiring emergency department (ED) visits, from 1.4 to 0.11 visits (*p* = 0.04). All patients reported a significant improvement in their quality of life after receiving lanadelumab. No severe attacks were reported with long-term lanadelumab prophylaxis. Furthermore, all patients remained well, and no adverse effects, including injection-site reactions, were reported after 9 months of treatment.

**Table 2:** Effect of lanadelumab on number of angioedema attacks per month after one month, two months and three months of treatment, and at the end of the study period

	Baseline	After 1 month	After 2 months	After 3 months	At the end of study period
**Extended treatment group**					
*Patient 1*	3	0	0	0	0
*Patient 4*	3.5	0	0	0	0
*Patient 7*	0.5	0	0	0	0.2
*Patient 8*	0.5	0	0	0	0
**Standard treatment group**					
*Patient 2*	4	0	0	0	0
*Patient 3*	4	0	0	0	0.11
*Patient 5*	12	0	0	0	0
*Patient 6*	20	5	3.5	3	1.26
*Patient 9*	6	0	0.5	0.33	0.33

## Extended dosing regimen

Four patients were started on lanadelumab every 4 weeks, as off-label use. As such, patients were closely monitored and reviewed every 2 weeks for the initial 8-week period, so that, where necessary, treatment interval shortening was carried out. There was a statistically significant improvement in number of attacks per month after lanadelumab (*p* = 0.03). Three patients achieved remission from attacks at the end of the study period. None of the patients required shortening of their treatment interval, as their disease was well-controlled. In fact, 2 patients missed their scheduled doses by 7 weeks and 8 weeks, respectively. Both patients did not experience any attacks during that period, and were re-commenced on a four-week regimen.

## Standard dosing regimen

Five patients were started on lanadelumab every 2 weeks. There was a statistically significant improvement in number of attacks per month after lanadelumab (*p* = 0.01). Two patients achieved complete remission from attacks at the end of the study period. We extended the treatment interval in three patients, as their disease was well-controlled for 3 months since initiation of lanadelumab. Two of the 3 patients were well-controlled and successfully switched to lanadelumab every 4 weeks, whereas the third patient had to be switched back to lanadelumab every 2 weeks due to inadequate control of angioedema attacks.

## Discussion

Our analysis shows that lanadelumab is an effective and well-tolerated prophylactic agent in difficult-to-treat HAE patients. All our patients reported a significant improvement in the number of angioedema attacks per month, which is in line with previous real-life reports.^1^^,^^8^^,^^9^ Moreover, over half of the patients experienced complete remission from attacks since starting lanadelumab. Patients also reported a significant improvement in their quality of life after starting lanadelumab. While this was not assessed in an objective manner, all our patients provided a global assessment of quality of life. Buttgereit et al formally assessed the effect of lanadelumab on quality of life in HAE patients, reporting a statistically significant improvement in the Angioedema Quality of Life (AE-QoL) scores after 30 weeks of treatment.^8^ Moreover, Iaboni et al reported that two-thirds of their patients reported no impact on social outings due to angioedema attacks, with only 1 patient having to miss work, after at least 6 months of lanadelumab.^9^

Current guidelines on lanadelumab in HAE suggest a starting dose of 300 mg every 2 weeks, and possible extension of the treatment interval to 4 weeks if disease is well-controlled.^7^^,^^11^^,^^12^ Four of our patients were started on lanadelumab 300 mg every 4 weeks, as off-label use. This was guided by the mild severity of their disease (less than 4 attacks per month), relative to our remaining patients. All 4 patients receiving lanadelumab every 4 weeks were well-controlled on this regimen, with 3 of these patients reporting no attacks since starting treatment. In addition, none of the patients required shortening of the treatment interval to 2 weeks, as their disease was well-controlled. This is an interesting finding, as it shows that some patients may achieve the same disease control, despite being on a longer treatment interval. An every-four-week regimen was used in the HELP trial, which showed that this regimen is effective in reducing angioedema attacks.^10^ To our knowledge, however, no real-life experiences reported the use of lanadelumab 300 mg every 4 weeks as a starting dose. Clinical trials are needed to explore if starting patients on an every-four-week regimen is comparable in efficacy to an every-two-week regimen, and whether there are factors that can guide the selection of one dosing interval over the other.

Five of our 9 patients were started on lanadelumab 300 mg every 2 weeks, according to current guidelines, and as dictated by their disease severity at baseline (4 or more attacks per month). A trial of treatment interval extension to 4 weeks was done in 3 patients, of which 2 responded well and were successfully switched to an every-four-week regimen. The third patient experienced an attack, and was therefore switched back to the original regimen. We were unable to extend treatment in the remaining 2 patients of this treatment group (n = 5), as one had a high BMI, and the other did not achieve adequate disease control. Buttgereit et al applied an interesting method of treatment interval extension, by increasing the two-week interval by 3 days at a time, after 1 or 2 months of well-controlled disease, depending on the presence or absence of attacks after starting lanadelumab.^8^ Using this method, most of their patient group achieved a treatment interval of 30 days. Further studies are needed to identify if patients can benefit from gradual treatment interval extension.

From a financial standpoint, there are implications to be considered when applying different lanadelumab regimens. The cost of 1 dose of lanadelumab is $20,538.^13^ By applying a basic calculation, the mean number of doses in our four-week group (n = 4) was 8.75 (SD 5.2), yielding an average cost of therapy of $179,707.5. On the other hand, the mean number of doses in our two-week group (n = 5) was 13.4 (SD 9.0), with the average cost of therapy being $275,209.2. While this is only an approximate extrapolation, if a four-week regimen is proven to be equal in clinical efficacy compared to a two-week regimen, this could potentially reduce costs for patients and insurance companies.

## Conclusion

Based on our findings, lanadelumab is an effective prophylactic medication in difficult-to-treat HAE patients. It remains unclear whether, in routine clinical practice, there is significant benefit in using a 300 mg two-week regimen versus a 300 mg four-week regimen. Our experience shows that there was no difference in the effect of lanadelumab on disease control between the 2 treatment groups. In fact, more patients achieved complete remission from attacks on the extended regimen compared to the standard regimen, although this may be due to the higher number of attacks at baseline in the two-week group. Our analysis also shows that an extended regimen can be more cost-effective for patients and insurance companies. Indeed, lanadelumab 300 mg every four weeks is off-label, and we do not recommend its use, until further studies are carried out. However, this regimen can be considered as an effective treatment in difficult-to-treat patients who could otherwise not afford this treatment at all. If patients do not show a complete response on a four-week regimen, shortening of dose intervals should be considered. Clinical trials are needed to directly compare the effectiveness of an extended versus a standard dosing regimen, and to identify patient-related factors that can be used to guide dosing frequency.

## Abbreviations

AE-QoL, Angioedema Quality of Life; BMI, Body Mass Index; C1-INH, C1 inhibitor; FDA, Federal Drug Administration; HAE, Hereditary Angioedema.

## Funding

This manuscript was funded by Charité – Universitätsmedizin Berlin as part of the DEAL agreement.

## Availability of data and materials

All data and materials are available upon request.

## Author contributions

OG collected patient data. MA, OG, and MM contributed to writing the manuscript. MA, MM, HA, FS, and DG reviewed the manuscript.

## Ethics approval

No actual work with human subjects or animals had been conducted in relation to this letter to the editor.

## Consent for publication

All authors have reviewed and approved of the final manuscript.

## Declaration of competing interest

None of the authors have any competing interests.
